# Centering and Racial Disparities (CRADLE study): rationale and design of a randomized controlled trial of centeringpregnancy and birth outcomes

**DOI:** 10.1186/s12884-017-1295-7

**Published:** 2017-04-13

**Authors:** Liwei Chen, Amy H. Crockett, Sarah Covington-Kolb, Emily Heberlein, Lu Zhang, Xiaoqian Sun

**Affiliations:** 1 Department of Public Health Sciences, Clemson University, Clemson, SC 29634 USA; 2 Department of Obstetrics and Gynecology, Greenville Health System, Greenville, SC 29605 USA; 3 Georgia Health Policy Center, Andrew Young School of Policy Studies, Georgia State University, Atlanta, GA 30303 USA; 4 Department of Mathematical Sciences, Clemson University, Clemson, SC 29634 USA

**Keywords:** Group prenatal care, Individual prenatal care, Preterm birth, Maternal behavioral factors, Maternal psychosocial factors

## Abstract

**Background:**

In the United States, preterm birth (PTB) before 37 weeks gestational age occurs at an unacceptably high rate, and large racial disparities persist. To date, medical and public health interventions have achieved limited success in reducing rates of PTB. Innovative changes in healthcare delivery are needed to improve pregnancy outcomes. One such model is CenteringPregnancy group prenatal care (GPNC), in which individual physical assessments are combined with facilitated group education and social support. Most existing studies in the literature on GPNC are observational. Although the results are promising, they are not powered to detect differences in PTB, do not address the racial disparity in PTB, and do not include measures of hypothesized mediators that are theoretically based and validated. The aims of this randomized controlled trial (RCT) are to compare birth outcomes as well as maternal behavioral and psychosocial outcomes by race among pregnant women who participate in GPNC to their counterparts in individual prenatal care (IPNC) and to investigate whether improving women’s behavioral and psychosocial outcomes will explain the potential benefits of GPNC on birth outcomes and racial disparities.

**Methods/design:**

This is a single site RCT study at Greenville Health System in South Carolina. Women are eligible if they are between 14–45 years old and enter prenatal care before 20 6/7 weeks of gestational age. Eligible, consenting women will be randomized 1:1 into GPNC group or IPNC group, stratified by race. Women allocated to GPNC will attend 2-h group prenatal care sessions according to the standard curriculum provided by the Centering Healthcare Institute, with other women due to deliver in the same month. Women allocated to IPNC will attend standard, traditional individual prenatal care according to standard clinical guidelines. Patients in both groups will be followed up until 12 weeks postpartum.

**Discussion:**

Findings from this project will provide rigorous scientific evidence on the role of GPNC in reducing the rate of PTB, and specifically in reducing racial disparities in PTB. Establishing the improved effect of GPNC on pregnancy and birth outcomes can change the way healthcare is delivered, particularly with populations with higher rates of PTB.

**Trial registration:**

NCT02640638 Date Registered: 12/20/2015.

## Background

### Preterm Birth

It is well known that preterm birth (PTB) occurs at unacceptably high rates within the United States (U.S.), where 9.6% of babies were born preterm at less than 37 weeks gestational age in 2015 [1]. Large racial disparities persist, particularly between White and Black women; the latter had a 13.4% preterm birth rate in 2015 [1]. PTB is the leading cause of newborn death and disability. Moreover, the racial disparities in the newborn period have the potential to contribute to disparities in chronic disease, academic achievement and economic opportunity across the lifespan [2–4]. To date, medical and public health interventions have achieved limited success in improving rates of PTB. The causes of poor birth outcomes and health disparities are complex, involving biological, behavioral, psychosocial, socio-demographic, environmental, and medical factors [2]. Interventions that do not address these factors in a comprehensive way will have limited success.

Prenatal care has been the foremost strategy to improve pregnancy and birth outcomes in the U.S. Increasing access to individual prenatal care (IPNC) in the last twenty years has marginally improved rates of PTB, but has not made any headway in reducing racial disparities in birth outcomes [5–7]. Most IPNC appointments are short (10–15 min) and focus more on triaging medical risks, with limited time for psychosocial interventions or health promotion [8, 9]. Women are often referred to childbirth education or ancillary services to address these needs [10]. Deviation from recommended prenatal care content is associated with PTB and low birth weight (LBW), indicating the importance of prenatal care models that are structured to provide comprehensive content [11]. In addition, psychosocial factors during pregnancy (e.g. stress, anxiety, depression, and coping responses) are gaining increased attention as critical contributing factors to poor birth outcomes; however, the current IPNC model does not adequately consider these factors [12]. Innovative models in prenatal care that can address not only physical risk assessment, but also women’s psychosocial wellbeing are therefore highly desired.

### CenteringPregnancy group prenatal care model

The CenteringPregnancy group prenatal care (GPNC) model is distinctly different from the traditional practice of IPNC, requiring a paradigm shift in health care delivery for obstetric healthcare providers and fundamental changes to the patient-provider interaction. In this model, developed and trademarked by the Centering Healthcare Institute (CHI) (Boston, MA), groups of 8–12 women who are all due to deliver in the same month receive their medical care in a series of ten two-hour group sessions which take place through the second and third trimesters [13, 14]. The group sessions begin with patients measuring their own weight and blood pressure, followed by an individual physical assessment from a credentialed health care provider (either a physician or a nurse practitioner/midwife). Once these assessments are complete, the group meets together for structured discussion and activities that follow the curriculum developed by the CHI. Group sessions also include time for socialization, which encourages group members to develop relationships with one another. Group session topics are organized by relevancy to gestational age (Table 1). The model allows the provider to adapt the content according to the needs and priorities of the participants, with deviations from the curriculum noted to be addressed at a later session. Women are encouraged to bring a support person (e.g., boyfriend, husband, friend, family member) for group sessions. CHI has established a site training and annual certification process to assure consistency and quality in implementation for obstetric practices offering GPNC [15]. This model has been implemented in several hundred practices in the U.S. [15].

**Table 1:** CenteringPregnancy group prenatal care (GPNC) curriculum outline

1	12–16 weeks	Prenatal testing, nutrition, healthy lifestyle choices	Exercise
2	16–20 weeks	Body changes in pregnancy, common discomforts, oral health	
3	20–24 weeks	Relaxation and stress reduction, breastfeeding	Gestational diabetes
4	24–28 weeks	Family relationships, violence and abuse, family planning, preterm labor	Breastfeeding
5	26–30 weeks	Labor, birth facility	Stress management/relaxation
6	28–32 weeks	The birth experience	
7	30–34 weeks	Newborn care	
8	32–36 weeks	Pregnancy to parenting transition, postpartum emotions, kick counts	Nutrition
9	34–38 weeks	Newborn safety, putting it all together	Preterm labor
10	36–40 weeks	Newborn care, growth and development, home and family changes	

There is promising evidence from both obstervational studies and radomized controlled trials (RCT) that suggests participation in GPNC is associated with reductions in preterm birth and low birth weight [16–19]. These findings have not been consistently reported in other studies [20–22]. The existing literature is limited by the small size of the previously randomized trials, as well as selection bias of the observational studies. Within this context, there is evidence that GPNC may result in reducing the racial disparity in preterm birth for Black women [16, 17]. Racial disparities in health have roots in inequalities in the provision of healthcare at the system level, the provider level, and the patient level. GPNC is designed to begin to address the racial disparities in all three levels. First, GPNC includes more patient-provider interaction time, with an average cumulative patient-provider interaction time of about 20 h throughout the pregnancy in GPNC, compared to 2 h for IPNC. More patient-provider interaction is believed to help develop the patients’ trust in their provider. Second, GPNC may reduce racial disparities in healthcare arising from unintentional prejudice and discrimination from healthcare providers, by allowing providers to develop a more robust understanding of patients’ family structures, social networks, employment or school, and physical home environment. Lastly, at the patient level GPNC is designed to promote goal setting and self-care of patients, empowering women to become more engaged in their health care and managers of their health by taking the responsibility for some of their health care.

### Current research gaps

Although this GPNC model has been successfully implemented in several hundred practices in the U.S., there are several significant gaps in the current literature. Existing studies are primarily observational studies that vary widely in quality, many of which are limited by self-selection bias and potential confounding [16, 18, 23, 24]. At the time we developed this proposal, only three RCTs had been published, but with mixed results. A secondary analysis of a study that was designed to examine GPNC’s impact on HIV risk behaviors and sexually transmitted infection demonstrated a 33% reduction in the odds of PTB in the GPNC group as compared with the control group [17]. The women in this study were younger than 25 years of age. A small trial in military settings did not find any significant difference in PTB or other birth outcomes [25]. The third trial in Iran also did not find any significant difference in PTB or LBW [26]. None of these studies were adequately powered to determine differences in rates of PTB, or the racial disparities in PTB in medically low risk women across all reproductive ages.

The mechanism by which GPNC may improve birth outcomes is unknown, but existing research suggests that GPNC contributes to positive psychosocial and/or behavioral changes in women [24]. Participation in GPNC has been associated with higher rates of adequate prenatal care, breastfeeding initiation, less excessive gestational weight gain, greater likelihood of using vitamin supplements during pregnancy, improved food security, and more knowledge for prenatal care and more prepared for labor and delivery [17, 19, 23, 25, 27–34]. Qualitative research indicates women generally find GPNC to be a positive experience, meeting many of their preferences for care [17, 19, 35–38]. Although these results are promising, no studies have evaluated whether improving these psychosocial and/or behavioral factors will explain the potential benefits of GPNC on racial disparities in birth outcomes.

The current study addresses the most significant gaps in the current GPNC literature: the lack of a rigorously designed clinical trial to assess the impact for GPNC on PTB and the racial disparity in preterm birth. This study will also measure GPNC’s effects on patient activation, engagement, stress, and health behaviors as they relate to birth outcomes and answer whether improving these maternal psychosocial and behavioral factors will explain the potential benefits of GPNC on birth outcomes or on racial disparities.

### Preliminary research

In 2012, we published a retrospective study of 316 women who participated in GPNC compared with 3,767 women in IPNC [16]. The adjusted odds ratio for PTB for women in GPNC care was 0.53 (95% CI: 0.34, 0.81) as compared to women in the IPNC. The racial disparity in PTB between Black women and White women was also diminished for women in GPNC: the Black-White difference of PTB was 1.0% in the GPNC and 2.4% in the IPNC group. In 2012–2013, we conducted a mixed methods prospective cohort study comparing the effectiveness of GPNC to IPNC on women’s psychosocial health [24, 33]. We recruited 248 women, retaining 89% of participants through pregnancy and 84% into the postpartum period. Women completed surveys at two points during pregnancy and once in the post-partum period. Among women with inadequate initial social support, GPNC participants scored 3.16 points lower (*P* = 0.03) on a scale of prenatal distress in late pregnancy. Among women with high initial prenatal distress, GPNC participants scored 7.96 points higher (*P* = 0.008) on a measurement of planning and preparation coping in late pregnancy [24].

Preliminary data from our retrospective and prospective studies demonstrate the potential for GPNC not only to reduce PTB, but also to reduce racial disparities in PTB. Our preliminary data also indicates that GPNC improves women’s psychosocial outcomes as well, particularly for those reporting high levels of stress in early pregnancy. By using a RCT design, this proposed study will reduce the possibility of selection bias and influences from potential confounding, and have adequate power to conclusively assess the efficacy of GPNC compared with IPNC on rates of PTB and racial disparities in birth and other outcomes among White and Black women.

### Specific aims and hypotheses

The proposed study will employ a RCT design adequately powered to detect difference in PTB by intervention and race. The study will be conducted in a large prenatal care provider in South Carolina (SC), with historically high PTB rates of 16.4%. The proposed study builds on an established research program focused on assessing the impact of GPNC vs. IPNC on birth outcomes and will investigate the following Specific Aims:

### Specific Aim 1

To compare the rate of PTB prior to 37 weeks gestational age and other selected birth outcomes and pregnancy complications (e.g. birth weight, caesarean section rate) among women who participate in CenteringPregnancy GPNC to their counterparts in IPNC.

#### Study hypothesis 1

Women who participate in GPNC will have a lower rate of PTB and other improved birth outcomes as compared to their counterparts in IPNC.

### Specific Aim 2

To compare the risk difference of PTB and other selected birth outcomes of Black women vs. White women in GPNC to the risk differences of Black women vs. White women in IPNC.

#### Study hypothesis 2

The risk difference of PTB and other selected birth outcomes between Black and White women in the GPNC group is smaller than that in the IPNC group.

### Specific Aim 3

To compare whether women in the GPNC have improved maternal psychosocial (i.e. activation, engagement, stress) and behavioral (i.e. smoking, healthy eating, health practices) outcomes as compared to their counterparts in IPNC and to explore whether improving certain maternal psychosocial and behavioral outcomes will explain the potential benefits of GPNC on racial disparities in birth outcomes.

## Methods/design

### Ethical approval

The study protocol (GHS Pro00043994) was approved by Institutional Review Board (IRB) of the Greenville Health System and Clemson University in June 2015. All the study participants will sign written consenting form before they are enrolled in the study.

### Study design and setting

This proposed study will employ an un-blinded RCT design to assess the efficacy of GPNC compared with IPNC on rates of PTB and racial disparities in birth outcomes among White and Black women. Eligible women will be recruited at the time of entry to prenatal care, and will be followed through delivery and for 12 weeks postpartum. All research activities will be conducted at the Greenville Health System (GHS) Obstetrics (OB) Center and ultrasound unit, located in Greenville, SC. Greenville is part of a metropolitan area with a population of approximately 640,000 people, and is approximately 80% urban and 20% rural [39, 40]. The practice has been serving women in the area for more than 40 years, with an annual delivery volume of approximately 2,750 women. The GHS OB Center first began offering GPNC in 2008, and has maintained annual site approval from the CHI since that time. To date, 3,633 patients have attended Centering groups at this practice.

### Patient population

The target population consists of medically low-risk pregnant women of different races/ethnicities. In our retrospective cohort study published in 2012, our study population in the comparison group included 45.8% non-Hispanic White, 25.5% non-Hispanic Black, 22.2% Hispanic and 6.5% “Other” race/ethnicity [21]. We expect that our current study population will be similar.

To be eligible for this study, women will 1) be between 14–45 years of age, and 2) enter prenatal care before their 20^th^ week of gestation. Study exclusion criteria include 1) medical complications of pregnancy which would preclude prenatal care provision by nurse practitioners or participation in group care (e.g. pre-gestational diabetes, severe chronic hypertension, active pulmonary tuberculosis, massive morbid obesity or severe psychiatric illness), 2) multiple gestation, 3) lethal fetal anomalies, and 4) low literacy. The exclusion criteria were set to limit the study participants as low-risk patients, which is consistent with both the GPNC model design and the scope of medical practice of nurse practitioners/nurse midwives who provide GPNC. Women who have participated in this study in a previous pregnancy will also be excluded, in order to maintain independence. Based on our preliminary data, application of these criteria will result in an estimated exclusion of 10% of women for late entry to care and 8% for pre-existing medical complications. The exclusion criteria will be applied to all women before randomization. We will track demographic characteristics and reasons for exclusion for women who are ineligible for the study as well as for those who are eligible but decline participation.

### Participant recruitment and screening

**Fig. 1:**
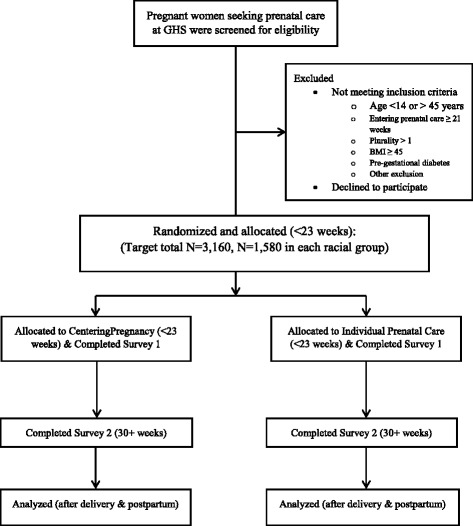
Flow chart of patient recruitment and follow up

We propose to recruit and follow 3,160 (*N* = 1,580 in each group) pregnant women (see section for sample size calculations). Approximately 250 women begin prenatal care at the GHS OB Center each month, and 75% of them will be eligible for the proposed study (180 eligible women per month). The provider will screen all new patients presenting to the practice for prenatal care (at the first prenatal visit) for eligibility and to introduce the study to eligible women. Given our previous recruitment rate of 50%, we estimate that the targeted study sample size will be achieved in 3 years. At the time of the nurse education visit at the GHS OB Center, all patients will be screened for study eligibility based on review of medical records and a series of screening questions answered by the GHS intake nurses. The final eligibility screening will take place after the patient is scheduled with a healthcare provider. This may be the same day, or may be a separate day from the nurse education visit. Once the history and physical exam has been completed, the research nurses will approach eligible patients for study enrollment. Patients will be taken to a quiet space to have the study explained. The enrollment process involves a face-to-face interview with one of the study team for verification of study eligibility and counseling regarding study procedures, potential benefits and risks, prior to obtaining written consent. Recruitment began in July 2016 and will be monitored monthly. Figure 1 describes the flow of participants through the study recruitment and data collection.

### Randomization and masking

#### Randomization

Women who are eligible for the study will be offered participation. Women who are interested in participation will have the opportunity to ask questions, confirm interest, provide consent, undergo group assignment, as well as complete the baseline data collection (Survey 1). Women who agree to participate will be randomized 1:1 to IPNC or GPNC based on computer-generated group assignment. These assignments will be stratified by race and ethnicity (Black, White, Hispanic, Mixed, and Other).

#### Masking

Due to the nature of the intervention, it is not possible to blind the group assignment to the participants and providers, but we will mask the providers to the study data. In addition, we will mask the data analysts regarding the group assignment to reduce bias.

### Interventions

#### GPNC (intervention) group

Women will be grouped with other women who are all due to deliver in the same month, and will receive their routine prenatal care together during ten 2-h group sessions meeting CHI’s established standards for CenteringPregnancy. Women will receive a patient notebook for their health information, educational materials, and activities to promote goal setting and self-care. During the first 30 min of each group session, women will measure and record their own weight and blood pressure, and then a credentialed healthcare provider (nurse practitioner, nurse midwife, or physician) will conduct a brief physical assessment in a semi-private area of the group space. Following the individual health assessments, the healthcare provider and a co-facilitator will lead the group discussion for the remaining 60–90 min of each session. Topics discussed throughout sessions include pregnancy and nutrition, childbirth preparation, exercise, stress management, relationships, and parenting (see Table 1). Women randomized to GPNC will have access to additional IPNC visits (outside of the ten scheduled group sessions) as needed to address complications that may arise between group sessions. The GPNC curriculum is designed to be culturally appropriate and the study site will deliver the intervention in both English and Spanish.

#### IPNC (control) group

Women will receive standard, traditional individual prenatal care in accordance with the schedule of visits recommended by the American College of Obstetricians and Gynecologists [41]. Women will attend monthly visits for the first 28 weeks of pregnancy, every two to three weeks until 36 weeks gestational age, then weekly until delivery. Visits will include ongoing physical assessment as well as patient education on common complications of pregnancy, childbirth preparation, breastfeeding, and other relevant topics as needed. Women randomized to IPNC will not attend GPNC sessions.

#### Both groups

Women will receive routine medical screenings as well as specialized tests, interventions, and referrals depending on risk factors and the course of pregnancy [41]. Women with a history of previous preterm birth who qualify for progesterone treatment will receive it [42].

### Compliance, cross-over, and follow-up

Aspects of prenatal care provision and participation will be assessed through medical record review and patient surveys, to measure compliance of both groups, including detailed data for each participant’s use of prenatal care services.

Women who have a miscarriage or leave the practice and deliver their baby at a different hospital system will be treated as lost to follow-up in the final analysis. However, we will make every effort to obtain data for women who deliver at other facilities by requesting medical records when we know the location of delivery, and by obtaining their birth outcomes data through the vital statistics database with the SC Department of Health and Environmental Control.

No matter the directions and the reasons of cross-over and regardless of the number of missed prenatal care visits, participants will be kept in their original assignments, as the primary analysis follows the intent-to-treatment approach. We will also do a secondary analysis measuring outcomes “as treated,” using patients who attend at least five visits of their assigned model of care.

### Retention plan

Retention of the research participants will be one of the most important tasks of this study. Retention begins at the time of recruitment, when our research team establishes a positive rapport with the research subjects. This includes providing information in a friendly manner, being responsive to their concerns, providing women with enough detail to understand the requirements of participation and stressing the importance of the potential research findings. Special efforts that will be taken to minimize the loss to follow-up include: 1) provide reminder text messages (will be made 3 days before each study visit), 2) limit participant burden by having recruitment and survey activities conducted when women are already at the OB Center, 3) provide child care for women attending the longer group care sessions, and 4) provide incentives/compensations. In our previous prospective studies, we have achieved an excellent retention rate: 92% at delivery and 80% at postpartum visits. We were able to accomplish this through extensive follow-up with patients via phone calls and text messages, friendly research staff, and incentives that increased in value as they went through the study. For this study, we are budgeting $75 per patient (in both groups) for incentives. We will offer gift cards at: completion of randomization and the Survey 1 ($25), attending 5 visits in the assigned group treatment and completion of the Survey 2 after 30 weeks gestational age ($50).

### Data collection

For this study, data will be collected at the following 3 time points: 1) At the time of Baseline Visit (8–23 weeks of gestational age), women will undergo group assignment and complete the baseline data collection (Survey 1); 2) After 30 weeks gestational age, research nurses will meet participants prior to or after their prenatal care appointment to administer the second survey (Survey 2); and 3) Postpartum data collection will be conducted after women attend their postpartum visit and consist of medical chart abstraction, and will not require patient contact. The study surveys will be administered electronically. Patients’ medical information will be collected from the electronic medical record (EMR). Regular data review/reports will be generated through the EMR to monitor enrollment, group balance, and the timing of the reminder calls & patient incentives. The overall timeline for study visits and data collection is summarized in Table 2.

**Table 2:** Data collection and outcome measurement

	Gestational age <23 week (Survey 1)	Gestational age 30+ week (Survey 2)	12-week Postpartum medical chart review
Birth outcomes and pregnancy complications			
Gestational age at delivery			X
Birth weight			X
APGAR scores			X
Admission to neonatal intensive care unit (NICU)			X
Intrauterine fetal demise			X
Neonatal death			X
Pre-eclampsia			X
Gestational hypertension			X
Gestational diabetes			X
Intrauterine growth restriction			X
Macrosomia (weight >4000 g.)			X
Hospital admission during pregnancy, reason			X
Induction and reason			X
Maternal behavioral and other measures			
Maternal smoking, alcohol and marijuana use	X	X	X
Physical activity, dietary intake, and multivitamin use		X	
Maternal psychosocial measures			
Patient activation measure (PAM)	X	X	
Prenatal planning and preparation coping		X	
Prenatal anxiety, and depressive symptoms	X	X	
Shift and Persist	X		
Discrimination in prenatal care		X	
Stress management		X	
Perceived family support	X		
Support from baby’s father & relationship with baby’s father		X	
Life stressor, financial stressor and housing instability in pregnancy		X	
Perceived stress and neighborhood safety	X		
Everyday discrimination scale	X		
Role of religious on dealing with stress	X		
Adverse childhood experience	X		
Interconception care			
Attendance at postpartum visit			X
Postpartum contraception use			X
Demographics			
Race and ethnicity	X		
Age, income, education, marital status, insurance, employment, household characteristics	X		
Reproductive history			
Pre-pregnancy weight			X
Previous preterm birth			X
Number of prior pregnancies, live births, and complications			X

### Study outcomes

*The primary study outcome* is PTB (defined as birth at less than 37 weeks gestation).

#### The other study variables are

1. Birth outcomes and pregnancy complications: For example, birth weight; APGAR score; admission to neonatal intensive care unit; intrauterine fetal demise; neonatal death; maternal anthropometric measures; gestational weight gain; pre-eclampsia; gestational hypertension; gestational diabetes; intrauterine growth restriction; macrosomia (birth weight > 4000 g); hospital admission during pregnancy and the reason; mode of delivery; induction and reason.
2. Maternal psychosocial measures: For example, a) Patient Activation Measure (13 items assessing confidence, knowledge, and skills in managing health and healthcare); b) Coping Inventory (15 items reflecting a range of coping strategies to manage preparing for a baby, adapted from established coping scales with additional items developed through focus groups and pilot testing with pregnant women) [43]; c) Prenatal Distress Questionnaire (a17-item scale assesses common worries and stressors during pregnancy, including concerns about labor and delivery, paying for the baby’s expenses, and managing work, relationships, and childcare) [44]; d) Perceived Stress Scale (a generalized measure assessing how overwhelmed people feel about their life circumstances [45, 46]; e) Stress Management Practices subscale of the Health Promoting Lifestyle Profile II [47, 48]; f) Prenatal Anxiety (13 items measures the emotional state of anxiety arising from women’s concerns about their pregnancy) [49]; g) Depression (The Center for Epidemiological Studies Depression Scale) [50]; h) Shift and Persist (14 item scale measures resilience) [51]; i) Everyday Discrimination Scale [52]; j) Discrimination in Prenatal Care (derived from PRAMS, Centers for Disease Control and Prevention, 2014]; k) Adverse Childhood Experience Study questions (adapted from the Behavioral Risk Factor Surveillance System questionnaire 2014 version) [53]; l) Support from Baby’s Father [54]; m) Perceived Family Support [55]; n) Role of religiosity in dealing with stress, and o) Perceived Neighborhood Safety.
3. Maternal behavioral and other measures: for example, maternal smoking, alcohol and marijuana use; pregnancy intention; physical activity, dietary intake and multivitamin use; housing instability, access to healthcare, food insecurity, income, breastfeeding at hospital discharge and postpartum visit.
4. Race/Ethnicity: Identifying the race of participants will be our most important quantitative measure. Indeed, the epidemiologic literature suggests that the categories of race and ethnicity reflect distinct social and environmental influences rather than actual genetic variations between populations [56–59]. We appreciate that race is more of a social than biologic construct and differences in health outcomes are likely due to the broader social and environmental differences observed between racial groups. Therefore, we will plan on using patient self-report of race and ethnicity, based on the 2020 U.S. Census categories. These categories will be used as our primary definition in the main analyses. Additionally, we will provide a narrative space for women to self-identify racial categories which do not fit neatly into the options provided, as well as questions about cultural heritage and country of origin. Finally, we will ask women about their socially assigned race and ethnicity (how an individual’s race/ethnicity is classified by others), since this has also been associated with differences in health outcomes [56, 60]. Such additional information will be used in exploratory and sensitivity analyses.
5. Other demographic information: For example, education, employment, marital status, income, and insurance.
6. Reproductive history: For example, pre-pregnancy weight, previous history of preterm birth, and numbers of live birth and prior pregnancies.
7. Interconception care: For example, attendance at postpartum visit, and postpartum contraception use.

### Prenatal care process evaluation

Aspects of prenatal care provision and participation will be assessed through patient survey 2, medical record review, and existing management information systems to measure fidelity of both models and women’s experiences with care. Detailed data for each participant’s use of prenatal care services, including timing of prenatal care entry, number of visits, GPNC or IPNC visit type, provider identifier, and continuity of provider, will be collected from medical charts and will be used to describe prenatal care use.

#### In survey 2, all patients will complete

1. A subset of questions from the Pregnancy Empowerment Scale: to assess women’s engagement in their health care and in making their pregnancy healthy [61];
2. Attendance of support people: Women will report who attended prenatal care with them in a supportive role and how often.

#### GPNC provision will be monitored additionally through

1. Perceived Cohesion Scale: This brief, four-question scale measures patients’ sense of belonging and satisfaction with their group;
2. Adherence to GPNC educational curriculum, group size, and continuity: GPNC facilitators and the Project Coordinator currently track curriculum topics covered across sessions, group composition, and measures of GPNC fidelity for at least one session per group. These will be copied as part of data collection for this study;
3. Annual fidelity review results: The OB Center participates in annual fidelity reviews with the Centering Healthcare Institute and has maintained high fidelity to the GPNC model since its first assessment in 2010. Fidelity reviews include a site visit by Centering Healthcare Institute faculty, record reviews, interviews, and observation. The OB Center’s fidelity results will be monitored through the study to assure key elements of the model are in place.

*IPNC provision will be monitored additionally through* medical charts review: IPNC’s educational and counseling topics will be assessed by reviewing medical charts of a randomly selected sample after women have delivered, stratified by provider.

### Mechanism for reporting adverse event

Minimal risk to the patient is associated with this study, since patients in both arms will receive prenatal care which meets or exceeds the standards set by the American College of Obstetricians and Gynecologists. We plan to report any adverse event to the IRB and the NIH as appropriate. An adverse event report form will be generated and used for collecting adverse events during the study period. Throughout the study, the Principal Investigators (PIs) (One PI is the Director and leading physician in the study site) will monitor the participants for adverse events. Events determined by the PIs to be unanticipated problems involving risks to subjects or others will be reported the IRB as in accordance with IRB policy. Adverse events that are determined by the PI to not be unanticipated problems involving risks to subjects or others will be reported per IRB policy at the time of continuing review. In addition, NIH will be notified of by the PIs of all adverse events.

### Research compliance and regulatory audit

The regular research compliance and regulatory audit of this study will be conducted at GHS and performed by Corporate Compliance. The scope of the audit will include a review of internal controls through inquiry and detailed sample-based testing of the following processes: registration, visit charge, research study charge capture and billing. The investigators’ research records and the corresponding approved Institutional Review Board (IRB) documents will be reviewed to strengthen awareness of regulatory requirements and improve the ethical conduct of research. A copy of research regulatory findings will be sent to the GHS IRB.

### Data management and data sharing

The Study data will be collected through RedCap (study specific survey data) and EPIC (clinical data), which will be maintained at GHS. Regular data review/reports will be generated through the RedCap and EPIC to monitor the patient enrollment, group balance, compliance, and the time to make the reminding calls & patient incentives. The study related raw data will be transferred to Clemson University (every 6 months) for further data merging and cleaning. The merged datasets will be stored and maintained at Clemson University during the study active period. A virtual machine (VM) will be allocated for hosting the data raw and computation. Clemson University Clemson Computing and Information Technology (CCIT) will be responsible for the security management of the VM. The Clemson University research team will be responsible for data merge and cleaning. The cleaned dataset will be shared with GHS team.

The Study recognizes the final National Institutes of Health’s Statement on Sharing Research Data (NIH Guide: February 26, 2003; Notice # NOT-OD-03032). This Statement recognizes that the rights and privacy of people who participate in NIH-sponsored research must be protected at all times including in the course of sharing data. The Statement further recognizes that the PIs are granted sufficient time in which to analyze and publish their primary research initiatives. To support NIH’s long-standing policy to share and make available to the public the results and accomplishments of the activities that it funds, the study team has developed the following data sharing plan and has been approved by NIH. Data will be shared with NIH according to NIH existing policy. Before any data is sent to NIH, all personal identifiers will be removed and a unique study number will be assigned that is different from the study ID number used by our team to add a second level of security to protect participant confidentiality. Data sets from different sources (i.e. laboratory, screening, questionnaire) will be created using SAS, with each participant’s data linked using the unique study ID. Each data set will be labeled. A data dictionary of variable labels and codes will also be provided. We propose the use of SAS data files with accompanying data dictionaries; however other data systems can be used to prepare these files if preferred (such as, EXCEL, SAS or STATA). In addition, before a public use data set is created, data staff will review all fields to ensure that there are no fields that might reveal the identity of a subject. For example, providing zip code, race, and date of birth on a subject may be used to reveal the identity of a subject if the combination is unique.

### Sample size

We calculated our sample size based on Aims 1 & 2 with the primary outcome of PTB. Based on the literature and our preliminary data, the rate of PTB was approximately 10-16% of women in the traditional care setting [16]. The odds ratio of comparing PTB in GPNC to IPNC was 0.67 in the Ickovics study and 0.53 in our own study [16, 17]. Our own work also suggests that the risk difference of having PTB between Black women and White women was 1.0% in the IPNC and 2.4% in the GPNC. Taken together, it is reasonable to assume that the relative risk of PTB in the IPNC group could range from 10% to 16%. For aim 1, assuming the proportion of PTB rate of 13%, our study will need *N* = 2,712 (1,356 in each group) to detect the relative risk of PTB in GPBC vs. IPNC of 0.70 with an alpha of 0.05 and a power of 90%. For aim 2, assuming the proportion of PTB rate of 0.02, our study will need *N* = 2,748 (1,374 in each group) to detect reduction in risk difference of 1.4% between Black and White women in GPNC vs. IPNC group with an alpha of 0.05 and a power of 90%. With an estimated 15% attrition rate, we will target recruiting 3,160 (*N* = 1,580 in each group) women for this study. Table 3 gives the estimated minimum number (in each group) required to achieve an alpha of 0.05 and a power of 80% or 90% to detect the risk ratio from 0.5 to 0.7 in Aim 1 and to detect the risk difference from 0.01 to 0.08, given the rate difference (P_0_) of preterm birth between Black women and White women at the IPNC group ranges from 2% to 3% in Aim 2, This sample size estimates that the proposed study will have 90% power to detect racial difference on PTB for Aim 2.

**Table 3:** Sample size and power calculations

	Power = 80%			Power = 90%		
Aim 1	RR = 0.50	RR = 0.60	RR = 0.70	RR = 0.50	RR = 0.60	RR = 0.70
P_0_ = 0.10	435	721	1,356	582	965	1,814
P_0_ = 0.13	326	540	1,014	436	723	1,356
P_0_ = 0.16	258	427	800	345	571	1,070
Aim 2	RD = 0.01	RD = 0.014	RD = 0.018	RD = 0.01	RD = 0.014	RD = 0.018
P_0_ = 0.02	2,319	1,027	526	3,103	1,374	704
P_0_ = 0.03	3,826	1,799	995	5,121	2,048	1,332
P_0:_ the RD of PTB in IPNC group; RR: relative risk						

Abbreviation: RD = risk difference

### Analytic plan

Primary analyses for Aims 1, 2, and 3 will be conducted using an intent-to-treat approach. Study participants will be retained in their original assignment groups after the randomization in the analysis regardless of the number of missed visits, the use of additional services (e.g., progesterone), or loss to follow-up.

1. *Aim 1 analysis plan*: comparison of primary and secondary outcomes for GPNC vs. IPNC. To test the hypothesis for aim 1 that women who participated in GPNC will have a lower rate of PTB and other selected birth outcomes as compared to their counterparts in IPNC, multivariate regression models (logistic regression if the outcome is a binary variable and linear regression if the outcome is a continuous variable) will be employed with the intervention assignment as the primary independent variable. We will test the null hypothesis of coefficient of main exposure variable (GPNC vs. IPNC) equals zero. Stratified analyses will be conducted (e.g. race/ethnicity, previous history of PTB) to compare the primary and other outcomes within sub-groups.
2. *Aim 2 analysis plan*: comparison of racial disparities of primary and secondary outcomes for GPNC vs. IPNC. To test the hypothesis for Aim 2 that the Black-White differences in PTB and other selected birth outcomes is smaller in the GPNC as compared with that in IPNC, we will apply analyses similar to Aim 1 but adding an interaction term of prenatal care (GPNC vs. IPNC) with race (White vs. Black). We will test the null hypothesis of coefficient of interaction term equals zero. For aim 2, only women identified themselves as either Black or White will be included in the primary analysis. Then, we will conduct a sensitivity analysis to see whether including women who self-identified race as “Hispanic ethnicity”, “Mixed race” and “Other race” will influence the primary analysis results. Such sensitivity analysis will be guided by the results from the sub-groups analysis in Aim 1.
3. *Aim 3 analysis plan*: comparison the effects of GPNC on maternal psychosocial (i.e. activation, engagement, stress) and behavioral (i.e. smoking, healthy eating, healthy practices) changes and exploring whether improving maternal psychosocial and health behavior outcomes will explain the potential benefits of the GPNC on racial disparities in birth outcomes. To address Aim 3, we will first test whether changes in maternal health behaviors or psychosocial factors differ by intervention and race. To do this, a similar analytic plan as used in Aim 1 & 2 will be applied, however, the dependent variables will be changes of health behaviors or psychosocial factors from baseline (~20 weeks) visit to late pregnancy (30–36 weeks). If we observe any significant differences in these health behaviors or psychosocial factors by intervention or race, the next step is to explore whether and to what extent the differences in the intermediate health behavior or psychosocial factors could explain the intervention effect or racial disparities of birth outcomes. To do so, the main multivariate regression models will be examined with and without adjustment for the intermediate health behavior or psychosocial variables.

### Statistical review and interim analyses

Statistical review of the study will be conducted by a statistician periodically during the intervention phase of the study. Interim analyses will be performed to assess outcomes and to decide the continuation, alteration of study design, or early termination, as appropriate. For each of the hypothesis tests considered in this proposal, we plan to monitor the change of the power after having a reasonable number of patients (e.g. 50%) in each group. In addition, some Monte Carlo simulations might be adopted to get more accurate power estimates. If the power reaches the desired level and is quite stable as more patients are included gradually, we might stop collecting new data. Such interim analyses will also be used to monitor the patients’ safety and data quality.

## Discussion

This is a protocol for a RCT evaluating whether CenteringPregnancy contributes to the reduction of PTB and other poor maternal and birth outcomes, and improves the racial disparity in PTB among medically low-risk pregnant women. Additionally, this study will investigate the potential mediating effects of maternal psychosocial and health behavior factors. Findings from this project potentially have two broad areas of action. First, findings will build an understanding of the role of GPNC in reducing the rate of PTB, and specifically in reducing racial disparities in PTB. Second, establishing the superiority of GPNC can change the way healthcare is delivered, particularly to medically underserved populations. Results will support public health efforts and future research opportunities designed to improve the quality and effectiveness of prenatal care services in promoting positive birth outcomes and reducing racial disparities.

## Abbreviations

CHI: Centering Healthcare Institute
EMR: Electronic medical record
GHS: Greenville Health System
GPNC: Group prenatal care
IPNC: Individual prenatal care
IRB: Institutional Review Board
LBW: Low birth weight
OB: Obstetrics
PTB: Preterm birth
RCT: Randomized controlled trial
SC: South Carolina
U.S.: United States
